# *In Vivo* Antimalarial Activity of *Polyalthia longifolia* (Annonaceae) Leaf Extract and Assessment of Its Formulated Oral Dosage Forms

**DOI:** 10.1155/2021/6519346

**Published:** 2021-11-23

**Authors:** Samuel Korsah, Stephen Yao Gbedema, Marcel Tunkungmen Bayor, Mariam El Boakye-Gyasi, Frederick William Akuffo Owusu, Arnold Donkor Forkuo

**Affiliations:** Department of Pharmaceutics, Faculty of Pharmacy and Pharmaceutical Sciences, College of Health Sciences, Kwame Nkrumah University of Science and Technology, Kumasi, Ghana

## Abstract

Plant medicine is commonly employed to treat malaria and other infections in Ghana. However, many of these phytomedicines have not been scientifically investigated to justify their use. This study therefore sought to investigate the antimalarial property of *Polyalthia longifolia* leaves and to formulate suitable dosage forms for ease of administration. A four-day antiplasmodial suppressive and curative study was conducted on ethanol extract of *P. longifolia* leaves (PLE) using *Plasmodium berghei* infected albino mice. Tablet and suspension dosage forms of PLE were formulated and evaluated for quality and stability. Statistically significant (*P* < 0.05) parasitaemia suppression (61.25%) and cure (58.78%) were achieved at a PLE dose of 100 mg/kg, and increases in hematological indices (*P* < 0.001) were also observed in the PLE-treated mice as compared to the untreated group. The tablets passed the tests for uniformity of weight, friability (<1%), hardness, disintegration (<15 minutes), and *in vitro* dissolution (>70% release in 45 minutes). The sedimentation volume, rheology, viscosity, and pH of the formulated suspension were within the official specifications. The dosage forms showed consistency in PLE content (85–105%) and no changes in physicochemical properties over the six months period of stability study. The *in vivo* antimalarial activity of PLE has been established and oral dosage forms that conformed to Pharmacopoeial standards are formulated for use in the management of malaria.

## 1. Introduction

Malaria is a parasitic disease caused by a protozoan of the plasmodium genus. Malaria, according to the World Health Organization (WHO), was responsible for approximately 229 million clinical cases in about 87 malaria endemic countries and averagely about 409,000 deaths globally in 2019 [1]. WHO African region accounted for 94% of this global malaria statistics in 2019 (215 million cases and 386,000 deaths). In sub-Saharan African countries, *Plasmodium falciparum* is the main parasite responsible for malaria infections which is transmitted through the bite of infected female Anopheles mosquitoes. Children under 5 years, pregnant woman, immune-compromised individuals, and nonimmune travelers are at higher risk of the infection [1, 2].

In recent times, efforts and strategies have been devised to reduce malaria burden and its effect on national and global health [3]. One of the greatest setbacks has been the ability of *P. falciparum* to easily develop resistance to antimalarial drugs. This has rendered many of the clinically available antimalarial drugs ineffective, leading to long periods of hospitalization and high malaria mortality rates. The search for more and effective antimalarial agents is therefore crucial and cannot be over emphasized [4].

Medicinal plants have been used since time immemorial for the management of various diseases worldwide [5, 6]. These plants were usually extemporaneously prepared as decoction and/or infusion and administered as and when needed. Currently many of these herbal products are being manufactured on commercial basis without much consideration for stability and the appropriateness of the dosage forms. The scientific community has identified this gab and as such, a few medicinal plants including *Ipomoea digitata, Capparis erythrocarpus,* and *Alstonia boonei* have been studied and formulated into appropriate dosage forms [7, 8].

Medicinal plants continue to serve as source for the development of many orthodox drugs; quinine (obtained from the Peruvian *Cinchona officinalis*) and artemisinin (from the Chinese *Artemisia annua*) are typical examples [6, 9]. A number of other plants including *Polyalthia longifolia* have also been reported of being used to treat malaria and other diseases such as helminthiasis, skin infections, duodenal ulcers, and diabetes in Ghana [10]. Gbedema et al. [11] have reported the *in vitro* antiplasmodial activity of *P. longifolia* against chloroquine-resistant strains of *Plasmodium falciparum*.

*P. longifolia* is an evergreen tree commonly found in tropical regions [12]. It is usually grown as ornamental tree on the streets of many cities and towns and as a windbreak in many homes [11]. This study therefore sought to investigate the *in vivo* antimalarial property as well as formulate suitable oral dosage forms of the leaves extract for ease of administration in malaria therapy.

## 2. Materials and Methods

### 2.1. Plant Collection

Fresh leaves of *Polyalthia longifolia* var. Pendula were obtained from the botanical gardens of Kwame Nkrumah University of Science and Technology (KNUST), Kumasi, Ghana, in February, 2019, with a voucher specimen (KNUST/HMI/2018/L020) in the Herbal Medicine Department's Herbarium. The leaves were shade dried for 2 weeks and powdered. The powdered sample (1000 g) was cold macerated with 4.5 litres of 70% ethanol for 72 hours. The extract was then filtered, concentrated into a syrupy mass using rotary evaporator (at 40°C), and dried in the oven at 40°C for 3 days.

### 2.2. Phytochemical Screening

Phytochemical components of *Polyalthia longifolia* leaf extract (PLE) were identified using conventional techniques outlined by Trease and Evans [13].

### 2.3. Ethical Clearance

The study protocol was approved by the Ethical Review Committee of the Department of Pharmacology, KNUST (no. PHARM/ETHIC/ET11/20).

### 2.4. Experimental Animals

Male Wistar rats (130–170 g) and BALB/c albino mice (28–30 g) were obtained from Noguchi Memorial Institute for Medical Research, University of Ghana, Legon.

#### 2.4.1. Rodent Parasite

*Plasmodium berghei* (NK 65) was received from the Noguchi Memorial Institute for Medical Research at the University of Ghana, Legon, Accra, and used in the experiment. Han Wistar rats (10) were infected with the *P. berghei* NK 65 strain and used as donors. The National Institute of Health Guidelines for Care and Use of Laboratory Animals (Directive 2010/63/EU) were followed in all the experimental procedures.

### 2.5. *In Vivo* Antimalarial Activity Assay

The *in vivo* antimalarial activity of PLE was assessed in BALB/c albino mice using Peter's four-day suppressive [14] and curative tests [5] after which the respective average parasitaemia and percentage suppressions were determined.

#### 2.5.1. Four-Day Antimalarial Suppressive Test

A total of 36 male Albino mice (BALB/c) were used in this study. The mice were weighed and six mice were randomly selected and assigned Group I (normal control group). The remaining 30 were inoculated intraperitoneally with 0.2 mL of the *P. berghei* (NK 65) infected blood on the first day (Day 0) and then randomly divided into five groups of six mice each. Three hours after infection, the drugs were administered to the groups (Table 1). Treatment was continued daily for four consecutive days after which 1 to 1.5 mL quantities of blood were collected from the animals and divided in equal volumes into 2 sets of appropriately labelled 2.0 mL microtubes (one set of tubes contained anticoagulant and the other was plain empty tubes). The blood samples were collected using 5 mL syringes and by cardiac puncture after anesthetizing the mice with 2, 2, 2 tribromoethanol (2.5% w/v) as described by [16].

#### 2.5.2. Parasitaemia Level Determination

Thin blood films were prepared, stained with 10% Giemsa working solution, and observed for infected red blood cells under a light microscope using 100× magnification objective lens. The percentage parasitaemia was determined by counting the parasitized cells out of 100 red blood cells in each microscopy field. The average parasitaemia from eight fields was determined and the respective percentage suppressions were calculated.

#### 2.5.3. Antimalarial Curative Test

A total of 36 male Swiss Albino mice were used in this study. The mice were weighed and six were randomly selected and assigned to Group I (control group). The remaining 30 were inoculated via intraperitoneal injection with 0.2 mL of the *P. berghei* (NK 65) infected blood on the first day (Day 0) and then randomly divided into five groups of six mice each (Table 1). On the fourth day (Day 3), thin blood films were prepared from blood samples taken from the tails of the mice in each group for parasitaemia determination after which the drugs were administered consecutively for four days (i.e., Day 3 to Day 6). Parasitaemia levels were again determined for each group at the end of the drugs treatment (Day 7) and used to calculate percentage parasite suppression of the groups.

### 2.6. Hematological and Biochemical Evaluation

Hematological indices and biochemical parameters were evaluated as described by Jahanbakhshi et al. [17].

### 2.7. *Polyalthia longifolia* Leaves Extract (PLE) Tablet Formulation

PLE tablets (each containing 0.277 g of PLE) were formulated using the following: PLE (13.85 g), polyvinylpyrrolidone (1.25 g), lactose (8.25 g), starch (1.75 g), magnesium stearate (0.25 g), and talc (0.25 g). The tablets were prepared using the wet granulation method. Excluding magnesium stearate and talc, all the ingredients were thoroughly mixed using geometric addition. Water was used as the granulating fluid to obtain a wet mass which was screened through a sieve of aperture 2360 *μ*m and oven dried at 60°C for an hour. The dried granules were screened with a sieve of aperture 1680 *μ*m. Magnesium stearate and talc were added to the granules and mixed thoroughly before compression [18]. The flow properties and compressibility of the granules obtained were assessed by determining its angle of repose, Carr's index, and Hausner ratio. The granules were subsequently compressed into 50 tablets using a tableting machine with die volume set to deliver 0.512 g of tablet. The compressed tablets were evaluated for weight uniformity, friability, hardness, disintegration, and dissolution characteristics as specified in the pharmacopeias [18–21].

### 2.8. Oral PLE Suspension Formulation

The PLE suspension (200 mL) was formulated using PLE (4.0 g), Tween 80 (0.2 mL), sodium carboxymethyl cellulose (1.0 g), sodium benzoate (2.0 g), syrup BP (2.0 mL), and water (q.s.). All the ingredients were triturated to form a smooth paste which was then diluted with 50 mL of distilled water. The pourable mass was then transferred into a bottle and made up to volume with distilled water [22].

### 2.9. Quality Assessment of PLE Suspension

Sedimentation volume, pH, viscosity, level of microbial contamination, drug content, and stability studies were carried out on the formulated suspension [23].

#### 2.9.1. Sedimentation Volume of PLE Suspension

The PLE suspension (100 mL) was transferred into a 100 mL measuring cylinder and the original volume (Vo = 100 mL) were recorded. The suspension was left to stand undisturbed for an hour after which the final or ultimate volume (Vu) of the sediment was recorded. The sedimentation volume (F) was expressed

(1) $$\begin{array}{c} F=\frac{\text{Vu}}{\text{Vo}}. \end{array}$$

#### 2.9.2. pH and Viscosity of Formulated PLE Suspension

The pH and viscosity of the formulated suspension were determined at room temperature using a digital pH meter and Brookfield viscometer (at 50 rpm), respectively [23].

#### 2.9.3. Level of Microbial Contamination of Formulated PLE Products

The pour plate method was used to assess the microbial quality of the PLE products using 20 mL quantities of Nutrient Agar, Sabouraud Agar, MacConkey Agar, Mannitol Salt Agar, and Bismuth Sulphite Agar. The media were boiled to melt and stabilized at 45°C in a water bath. The formulated PLE suspension (1.0 mL) was added to each of the media. After thorough mixing, the contents were transferred into separate Petri dishes and allowed to solidify. The PLE tablets (10 tablets) were aseptically placed in 20 mL of sterile normal saline and suspended with the exception of Sabouraud agar (which was incubated at 25°C for 72 hours); all the plates were incubated in an inverted position at 37°C for 24 hours. After incubation, the colonies on the media were counted [24].

#### 2.9.4. Assay of Formulated Suspension

The formulated suspension (5 mL) was diluted with 0.1 M HCl to 100 mL. The resulting solution was filtered into a volumetric flask and made up to 200 mL with enough 0.1 M HCl, after which the absorbance was recorded at 315 nm. Nine replicate absorbance determinations were done and the mean was calculated.

### 2.10. Stability Studies of Formulated Products

Real-time and accelerated stability studies were carried out on the formulated tablets and suspension. At 3 and 6 months, drug content, hardness, and *in vitro* dissolution studies were evaluated for the formulated tablets whereas sedimentation volume, flow rate, viscosity, and pH were evaluated for the formulated suspension. This was done in accordance with the guidelines of the International Conference of Harmonization (ICH).

## 3. Results and Discussion

### 3.1. Phytochemical Composition of PLE

Plants contain phytochemicals responsible for the plant's antioxidant, antimicrobial, antiplasmodial, and antiparasitic activities. Tannins, flavonoids, sterols, triterpenoids, glycosides, alkaloids, and coumarins were present in PLE. Amelo et al. [25] reported that alkaloids, tannins, and flavonoids may possess antiplasmodial activity.

### 3.2. Peter's Four-Day Suppressive and Curative Test

Antiplasmodial agents with high chemosuppressive action inhibit the growth of merozoites. The parasitaemia suppression of the PLE-treated group was dose-dependent in this test (Table 2). Extracts with a suppression percentage equal to or more than 50% are regarded to have good therapeutic efficacy [15].

PLE at selected doses of 150 mg/kg, 100 mg/kg, and 12.5 mg/kg (Table 3) can be said to have interrupted with the asexual multiplication of the sporozoites in the hepatic cells.

### 3.3. Rane's Curative Test

An antiplasmodial agent with high curative efficacy kills trophozoites in the blood or prevents the development of gametocytes, preventing reinfection and subsequent transmission.

PLE had curative efficacy at the stated doses, confirming the plant's traditional use in the treatment of malaria (Table 4).

### 3.4. Effect of PLE on Hematological Indices of *P. berghei* Passaged Mice

A study by Kotepui et al. [26] indicated malaria as one of the conditions that leads to anemia, thrombocytopenia, and leukocytosis or leucopoenia. The PLE-treated groups reversed these reductions in a dose-dependent manner (Table 3).

### 3.5. Effect of PLE on Liver Status of *P. berghei* Passaged Mice

PLE did not have any untoward effect on the experimental animals (Table 5).

### 3.6. Effect of PLE on the Lipid Profile of *P. berghei* Passaged Mice

According to the results of the study (Table 6), the normal control, PLE-treated groups, and Artesunate-treated groups had significantly lower TC, LDL, and triglycerides than the untreated group. In contrast to the untreated group, the treated and normal groups had significantly higher HDL levels. This might be owing to a parasitized host's lipoprotein lipase system being impaired as a result of increased tissue lipolysis, which raises cholesterol and triglyceride production [27].

### 3.7. Effect of PLE on the Kidney Status of *P. berghei* Passaged Mice

The key markers in diagnosing renal damage are serum urea and creatinine. The study's findings point to PLE's potential to restore electrolyte balance and renal biomarkers. This suggests that PLE has the potential to have renocurative or renoprotective effects on malaria-induced kidney injury (Table 7).

### 3.8. PLE Granules Flow Properties and Formulated Tablet Quality

The granules exhibited excellent flow which makes it suitable for compression into a tablet (Table 8).

The amount of active ingredient(s) in tablets may vary when even distribution of the ingredients is not achieved during the granulation process. Also, uneven flow of granules into the die during punching of the tablets can also lead to significant varying weights of the resulting tablets. Significant disparity in tablet weights will affect dosing of such drugs. In this study, the mean tablet weight was 553 mg (Table 9) with a ±0.024 variation (±4.34% disparity) which is within the acceptable limit as specified in the BP (2018) [19]. The formulated PLE tablets stand the chance of delivering the appropriate doses when dispensed and administered. The tablets also displayed an average disintegration time of 11.50 ± 0.577 minutes which was within the BP (2018) specification of 15.00 minutes for immediate-release tablets. The average hardness and friability of the tablets (Table 9) fell within the acceptable limit [20].

### 3.9. Dissolution Profile of PLE Tablets

**Figure 1:**
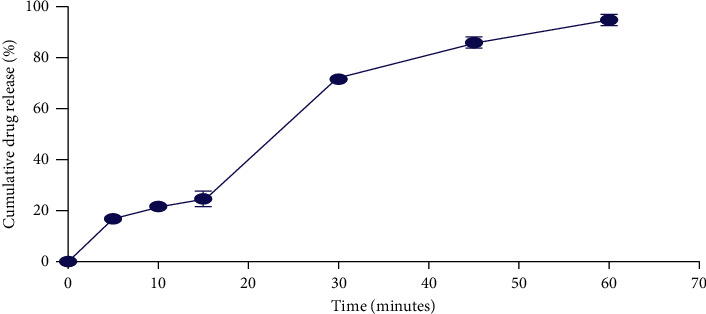
*In vitro* dissolution profile of PLE tablets.

The drug release from the formulated tablets was 87.02% at 45 minutes indicating a good release of the extract from the tablets (Figure 1) and complied with the British Pharmacopeia [19] limits for *in vitro* drug release for immediate-release tablets.

#### 3.9.1. The Quality of Formulated PLE Suspension

The viscosity of a suspension is of great importance for its stability and pourability. Based on the viscosity recorded for the formulated suspension (Table 10), its stability and pourability will not be adversely affected on storage. In a formulated drug product, the bioavailability and stability of the active ingredient are affected by the pH of the dosage form.

Extreme pH (high or low) can increase the rate of degradation of a drug [18]. The mean pH of the formulated suspension shows that the composition of the suspension can be well maintained upon storage. The percentage of drug content also fell within the acceptable limit of 85–105% (Table 10) as stipulated in [19, 20].

#### 3.9.2. Sedimentation Volume of Formulated Suspension

The sedimentation volume and sedimentation rate of the formulated suspension were within acceptable limits (Table 11), indicating that the formulation can be easily redispersed after settling which will maintain the accuracy in dosing when dispensed [18].

#### 3.9.3. Microbiological Quality of Formulated PLE Tablets and Suspension

Microbial contamination can cause chemical or physical changes in a product resulting in its spoilage. The total aerobic bacteria and fungi count were within the acceptable limits while harmful organisms such as *Staphylococcus*, *E. coli,* and *Salmonella typhi* were absent in the products (Table 11). The products have passed the microbiological quality assurance tests as prescribed in the British Pharmacopoeia (2018) [19].

### 3.10. Stability Studies

**Figure 2:**
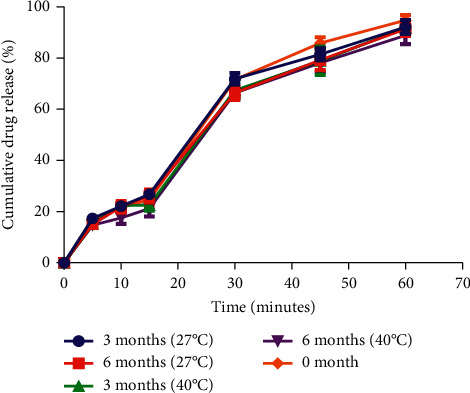
*In vitro* dissolution profile of formulated tablets at different temperatures.

The friability, hardness, and *in vitro* dissolution profile of the formulated tablets remained within the acceptable range at 27°C and 40°C for the period of the study (Table 9 and Figure 2). This connotes the products ability to remain safe and maintain its effectiveness throughout its shelf life. The pH, viscosity, drug content, sedimentation rate, and sedimentation volume were within the acceptable range over the period of study (Table 12). This indicates the consistency in redispersibility and accuracy in dosing of the suspension within the shelf life of the product.

## 4. Conclusion

*Polyalthia longifolia* leaves ethanol extract exhibited antiplasmodial activity against *Plasmodium berghei,* ANKA strain, and was able to restore hematological indices and some biochemical parameters altered by *P. berghei.* The ethanolic extract of *Polyalthia longifolia* leaves can be made into immediate-release tablets and suspensions that meet pharmacopoeial requirements and can be used as a replacement for antimalarial drugs.

## Figures and Tables

**Table 1:** Grouping of mice for the antimalarial study.

Groups	Description	Curative test
	Four-day suppressive test	
I	No inoculation; no treatment (NI)	No inoculation; no treatment (NI)
II	Infected; no treatment (NC)	Infected; no treatment (NC)
III	Infected; treated with 12.5 mg/kg PLE	Infected; treated with 100 mg/kg PLE
IV	Infected; treated with 50 mg/kg PLE	Infected; treated with 300 mg/kg PLE
V	Infected; treated with 150 mg/kg PLE	Infected; treated with 500 mg/kg PLE
VI	Infected; treated with 4.0 mg/kg of Artesunate	Infected; treated with 4.0 mg/kg of Artesunate

**Table 2:** Antiplasmodial four-day suppressive test of PLE in *P. berghei* passaged mice.

Groups	Dose (mg/kg)	Mean ± SEM	
		Level of parasitaemia	% suppression
I	NI	0.00	NA
II	NC	23.74 ± 2.860	NA
III	12.5	10.02 ± 1.244^*∗∗∗*^	50.25
IV	100	9.30 ± 1.126^*∗∗∗*^	61.25
V	150	8.36 ± 1.187^*∗∗∗*^	65.17
VI	4.0 (Artesunate)	6.34 ± 0.715^*∗∗∗*^	73.58

Results are presented as mean ± SD; *n* = 6. ^*∗∗∗*^*p* < 0.0001. ^*∗*^Values are *p* < 0.05 compared to negative control; ^a^ values are *p* < 0.05 compared to day 3. NI = normal control, NC = untreated group, and NA=not applicable.

**Table 3:** Effect of PLE on hematological parameters of *P. berghei* passaged mice.

Groups	Hematological indices					
	PCV (%)	MCV(FL)	Hb(g/dL)	RBC (× 10^6^/*µ*L)	WBC (× 10^3^/*µ*L)	Plat. (× 10^3^/*µ*L)
I	58.4 ± 0.441^a^	57.2 ± 0.441^a^	12.2 ± 0.318^a^	10.2 ± 0.321^a^	16.3 ± 0.288^a^	1244 ± 2.082^a^
II	20.3 ± 0.665	41.0 ± 0.441	6.5 ± 0.321	4.95 ± 0.326	4.9 ± 0.321	102 ± 1.202
III	48.0 ± 0.416^a^	57.9 ± 0.466^a^	13.0 ± 0.272^a^	8.29 ± 0.300^a^	8.2 ± 0.202^a^	249 ± 1.764^a^
IV	50.1 ± 0.441^a^	58.8 ± 0.521^a^	12.4 ± 0.352^a^	8.52 ± 0.511^a^	9.9 ± 0.261^a^	693 ± 0.881^a^
V	50.4 ± 0.665^a^	56.4 ± 0.696^a^	13.2 ± 0.202^a^	8.94 ± 0.459^a^	10.3 ± 0.191^a^	824 ± 1.202^a^
VI	55.1 ± 0.665^a^	55.1 ± 0.521^a^	12.1 ± 0.405^a^	8.37 ± 0.385^a^	13.1 ± 0.208^a^	927 ± 1.528^a^

Results are presented as mean ± SD (*n* = 6) of triplicate determinations; mean values with superscript “a” in column are considered significant at *p* < 0.001. Plat. = platelets.

**Table 4:** Antiplasmodial curative test of PLE in *P. berghei* passaged mice.

Groups	Dose (mg/kg)	Mean ± SEM		
		Level of parasitaemia		% suppression
		Day 3	Day 7	
I	NI	0.00	0.00	—
II	NC	20.36 ± 1.94	62.40 ± 8.38	—
III	100	30.18 ± 6.19	25.72 ± 0.38^a^∗^^	58.78
IV	300	29.32 ± 5.32	21.60 ± 2.49^a^∗^^	65.38
V	500	21.86 ± 1.51	17.88 ± 2.35^a^∗^^	71.35
VI	100 (Artesunate)	18.38 ± 0.74	4.30 ± 0.41^a^∗^^	93.11

Results are presented as mean ± SD; *n* = 6. ^*∗∗∗*^*p* < 0.0001.  ^*∗*^Values are *p* < 0.05 compared to negative control; ^a^ values are *p* < 0.05 compared to day 3. NI = normal control, NC = untreated group, and NA=not applicable.

**Table 5:** Effect of PLE on liver status of *P. berghei* passaged mice.

Tests	Groups					
	I	II	III	IV	V	VI
AST (U/L)	51 ± 1.009^a^	220 ± 1.10 0	80 ± 1.030^a^	73 ± 1.070^a^	65 ± 1.008^a^	59 ± 0.5000^a^
ALT (U/L)	40 ± 1.000^a^	120 ± 0.500	50 ± 0.500^a^	40 ± 1.000^a^	45 ± 0.500^a^	40 ± 1.000^a^
ALK phosphate (U/L)	113 ± 0.500^a^	174 ± 1.000	124 ± 0.500^a^	108 ± 1.000^a^	116 ± 1.000^a^	108 ± 0.500^a^
Bilirubin (total) (umol/L)	7.6 ± 0.150^a^	16.5 ± 0.300	13.3 ± 0.150^a^	12.7 ± 0.300^a^	10.4 ± 0.200^a^	9.6 ± 0.150^b^
Bilirubin (direct) (umol/L)	0.1 ± 0.050^a^	0.6 ± 0.040	0.2 ± 0.055^a^	0.2 ± 0.030^a^	0.1 ± 0.035^a^	0.1 ± 0.015^a^
Total protein (g/L)	56 ± 0.500^a^	22 ± 1.000	51 ± 1.000^a^	68 ± 0.500^a^	66 ± 1.450^a^	67 ± 0.900^a^
Albumin (g/L)	45 ± 1.000^a^	17 ± 0.500	29 ± 0.500^a^	37 ± 1.00^a^	37 ± 0.500^a^	29 ± 0.450^a^
Globulin (g/L)	30 ± 0.500^a^	16 ± 0.650	34 ± 0.650^a^	32 ± 0.700^a^	39 ± 0.700^a^	38 ± 0.600^a^

Results are presented as mean ± SD (*n* = 6) of triplicate determinations; mean values with different letters as superscript in column where “a” is considered significant at *p* < 0.001 and “b” is considered significant at *p* < 0.01.

**Table 6:** Effect of PLE on lipid profile of *P. berghei* passaged mice.

Test	Groups					
	I	II	III	IV	V	VI
Cholesterol (total) mmol/L	1.0 ± 0.057^c^	1.6 ± 0.029	1.1 ± 0.3150^c^	0.9 ± 0.050^c^	1.0 ± 0.115^c^	1.2 ± 0.045^c^
HDL (cholesterol) mmol/L	0.6 ± 0.005^a^	0.2 ± 0.015	0.5 ± 0.025^b^	0.5 ± 0.020^b^	0.7 ± 0.030	0.8 ± 0.070^a^
LDL (cholesterol) mmol/L	0.13 ± 0.025^a^	0.72 ± 0.035	0.14 ± 0.005^a^	0.13 ± 0.015^a^	0.10 ± 0.025^a^	0.13 ± 0.020^a^
Triglycerides mmol/L	0.6 ± 0.025^a^	1.5 ± 0.035	1.0 ± 0.005^a^	0.6 ± 0.015^a^	0.5 ± 0.025^a^	0.6 ± 0.020^a^

Results are presented as mean ± SD (*n* = 6) of triplicate determinations; mean values with different letters as superscript in column where “a” is considered significant at *p* < 0.001, “b” at *p* < 0.01, and “c” at *p* < 0.05.

**Table 7:** Effect of PLE on kidney status of *P. berghei* passaged mice.

Test	Groups					
	I	II	III	IV	V	VI
Urea mmol/L	5.4 ± 0.233^a^	10.7 ± 0.2404	7.8 ± 0.463^a^	8.8 ± 0.409^c^	7.6 ± 0.4726^a^	6.2 ± 0.2603^a^
Creatinine mmol/L	60 ± 1.453^a^	120 ± 0.8819	57 ± 0.8819^a^	66 ± 0.8819^a^	50 ± 0.8819^a^	62 ± 0.8819^a^

Results are presented as mean ± SD (*n* = 6); mean values with different letters as superscript in column where “a” is considered significant at *p* < 0.001 and “c” at *p* < 0.05.

**Table 8:** Flow properties of PLE granules.

Granule parameters	Values
Hausner ratio	1.04
Carr's index	4.29
Angle of repose	25.6

**Table 9:** Quality and stability analysis of PLE tablets.

PLE tablets	0.277 g PLE content (%)	Friability (%)	Hardness (N)	Disintegration time (min.)	Weight uniformity (g)
Initial assessment	—	0.27	53.56 ± 0.07	11.50 ± 0.577	0.5530 ± 0.024
Storage at 27°C for 3 months	96.83 ± 0.32	0.28	53.37 ± 0.10	—	—
Storage at 40°C for 3 months	91.50 ± 0.25	0.33	52.60 ± 0.15	—	—
Storage at 27°C for 6 months	93.38 ± 0.48	0.32	53.07 ± 0.09	—	—
Storage at 40°C for 6 months	89.60 ± 0.37	0.65	52.29 ± 0.08	—	—

**Table 10:** Flow rate, pH, viscosity, and sedimentation rates of PLE suspension.

Test	Flow rate (mLs^−1^)	pH	Viscosity (mPa)	Drug content (%)	Sedimentation volume (mL)	Sedimentation rate (mL/s)
Average	0.451 ± 0.072	6.40 ± 0.096	655 ± 0.098	99.1 ± 0.023	0.99	0.017

**Table 11:** Microbiological quality of PLE tablets and suspension.

Presence of microbial growth in media	PLE tablets result	PLE suspension result	Specification (BP 2018)
Total aerobic count on nutrient agar	2.1 × 10^1^ cfu/g	8.0 × 10^1^ cfu/mL	≤1.0 × 10^4^ cfu/g (mL)
*E. coli* on MacConkey agar	None detected	None detected	Absent (in 1 g/mL)
*Staph. aureus* on mannitol salt agar.	None detected	None detected	Absent (in 1 g/mL)
*S. typhi* on bismuth sulphite agar	None detected	None detected	Absent (in 1 g/mL)
Total yeast/mould count on Sabouraud agar	3.0 × 10^1^ cfu/g	1.0 × 10^1^ cfu/mL	≤1.0 × 10^2^ cfu/g (mL)

**Table 12:** Stability results of PLE suspension.

PLE suspension storage period	Flow rate (mL/s)	pH	Viscosity (mPa)	Drug content (%)	Sedimentation volume (mL)	Sedimentation rate (mL/s)
3 months (27°C)	0.453 ± 0.013	6.38 ± 0.075	648 ± 0.033	97.9	0.98	0.0286
3 months (40°C)	0.453 ± 0.013	6.37 ± 0.029	632 ± 0.027	96.9	0.98	0.0214
6 months (27°C)	0.450 ± 0.021	6.77 ± 0.062	643 ± 0.010	97.2	0.98	0.0321
6 months (40°C)	0.449 ± 0.031	6.80 ± 0.026	643 ± 0.056	95.3	0.97	0.0429

## Data Availability

The data used to support the findings of this study are included in the article and are also available from the corresponding author upon request.
